# Evidence for Cognitive Decline in Chronic Pain: A Systematic Review and Meta-Analysis

**DOI:** 10.3389/fnins.2021.737874

**Published:** 2021-09-22

**Authors:** Xueying Zhang, Rui Gao, Changteng Zhang, Hai Chen, Ruiqun Wang, Qi Zhao, Tao Zhu, Chan Chen

**Affiliations:** ^1^Department of Anesthesiology and Translational Neuroscience Center, West China Hospital, Sichuan University, Chengdu, China; ^2^Precision Medicine Research Center, West China Hospital, Sichuan University, Chengdu, China

**Keywords:** chronic pain, cognitive decline, memory, dementia, neuropathology

## Abstract

**Background:** People with chronic pain (CP) sometimes report impaired cognitive function, including a deficit of attention, memory, executive planning, and information processing. However, the association between CP and cognitive decline was still not clear. Our study aimed to assess the association of CP as a risk factor with cognitive decline among adults.

**Methods:** We included data from clinical studies. Publications were identified using a systematic search strategy from PubMed, Embase, and Cochrane Library databases from inception to October 10, 2020. We used the mean cognitive outcome data and the standard deviations from each group. The standardized mean difference (SMD) or odds ratio (OR), and 95% confidence intervals (CI) were performed for each cognitive decline outcome. *I*^2^-values were assessed to quantify the heterogeneities.

**Results:** We included 37 studies with a total of 52,373 patients with CP and 80,434 healthy control participants. Because these studies used different evaluative methods, we analyzed these studies. The results showed CP was associated with cognitive decline when the short-form 36 health survey questionnaire (SF-36) mental component summary (SMD = −1.50, 95% CI = −2.19 to −0.81), the Montreal cognitive assessment (SMD = −1.11, 95% CI = −1.60 to −0.61), performance validity testing (SMD = 3.05, 95% CI = 1.74 to 4.37), or operation span (SMD = −1.83, 95% CI = −2.98 to −0.68) were used. However, we got opposite results when the studies using International Classification of Diseases and Related Health Problems classification (OR = 1.58, 95% CI = 0.97 to 2.56), the Mini-Mental State Examination (SMD = −0.42, 95% CI = −0.94 to 0.10; OR = 1.14, 95% CI = 0.91 to 1.42), and Repeatable Battery for the Assessment of Neuropsychological Status memory component (SMD = −0.06, 95% CI = −0.37 to 0.25).

**Conclusion:** There may be an association between CP and the incidence of cognitive decline when some cognitive, evaluative methods were used, such as short-form 36 health survey questionnaire, Montreal cognitive assessment, performance validity testing, and operation span.

## Introduction

Pain is a multidimensional experience that includes sensory discrimination, emotional motivation, and cognitive evaluation interacting with each other (Treede et al., 2019). And chronic pain (CP) refers to the persistent pain after healing, which is considered to have occurred or exist without tissue damage. Unfortunately, millions of people in today's world are debilitated by various CP types such as headaches, arthritis, and backache. Chronic pain is a common, complex, and distressing problem that has a major impact on society and individuals. The 2016 Global Burden of Disease Study highlighted that pain and pain-related diseases were major contributors to the global burden of disability and disease (GBD Disease and Injury Incidence and Prevalence Collaborators, 2017). The annual costs of CP in direct medical costs are very high, and the loss of productivity it brings is huge. Therefore, pain relief is an important topic in clinical work and scientific research. Moreover, increased studies have shown that CP had many adverse outcomes, such as mood disorder, weight loss, daily functional loss, lower quality of life, and higher costs of health (Saraiva et al., 2018). Interestingly, CP seems to cause changes in cognitive function, which is another critical problem.

Cognitive decline could involve cognitive impairment in one or several areas, such as learning and memory, executive function, general cognitive functioning, attention, and social cognition (Sachdev et al., 2014). Moreover, cognitive decline has been reported to significantly impact medical compliance, workability, interpersonal interaction, and quality of life. Clinical studies have shown that CP was related to attention, memory, executive planning, and information processing (Berryman et al., 2013, 2014; Mazza et al., 2018). A longitudinal study of elders found persistent pain was associated with memory decline (Whitlock et al., 2017). A meta-analysis of longitudinal studies showed that headache was associated with a higher risk of dementia (Wang et al., 2018). However, another meta-analysis contained 10 prospective longitudinal studies that showed persistent pain was not associated with the incidence of cognitive decline (De Aguiar et al., 2020). A study also demonstrated pain was not associated with incident cognitive impairment in any of the three cognitive domains evaluated (attention, memory, and executive functioning) (Van Der Leeuw et al., 2018a).

The relation between pain and cognitive decline is complex, and pain may impair cognition by some mechanisms (Moriarty et al., 2011). Some studies suggested that the pain was associated with brain plasticity and structural changes in different cortical regions associated with learning, memory, fear, and emotional responses (Mazza et al., 2018). In addition, Moriarty et al. (2011) demonstrated that pain and cognition might share some common transduction pathways. Because pain is treatable, defining whether it is a risk factor of cognitive decline can promote targeted screening, preventive, and therapeutic interventions. Therefore, we applied a systematic review and meta-analysis to determine the evidence that CP is associated with cognitive decline.

## Methods

### Search Strategy

We searched the PubMed, Embase, and Cochrane Library from inception to October 10, 2020. Each database was searched separately. We searched synonyms of “chronic pain” and “cognitive decline.” Synonyms for “chronic pain” included “Chronic Pains,” “Pains, Chronic,” “Pain, Chronic,” “Widespread Chronic Pain,” “Chronic Pain, Widespread,” “Chronic Pains, Widespread,” “Pain, Widespread Chronic,” “Pains, Widespread Chronic,” and “Widespread Chronic Pains.” Synonyms for “Cognitive Decline” included “Cognitive Dysfunctions,” “Dysfunction, Cognitive,” “Dysfunctions, Cognitive,” “Cognitive Impairments,” “Cognitive Impairment,” “Impairment, Cognitive,” “Impairments, Cognitive,” “Mild Cognitive Impairment,” “Cognitive Impairment, Mild,” “Cognitive Impairments, Mild,” “Impairment, Mild Cognitive,” “Impairments, Mild Cognitive,” “Mild Cognitive Impairments,” “Mild Neurocognitive Disorder,” “Disorder, Mild Neurocognitive,” “Disorders, Mild Neurocognitive,” “Mild Neurocognitive Disorders,” “Neurocognitive Disorder, Mild,” “Neurocognitive Disorders, Mild,” “Cognitive Decline,” “Cognitive Declines,” “Decline, Cognitive,” “Declines, Cognitive,” “Mental Deterioration,” “Deterioration, Mental,” “Deteriorations, Mental,” “Mental Deteriorations,” “Cognitive disorders,” “Disorder, Cognition,” “Disorders, Cognition,” “Dementia,” and “Dementias.” Citations related to cognitive decline and CP were retrieved and exported to ENDNOTE, where duplicates were removed, and articles were reviewed. This systematic review and meta-analysis were followed according to Cochrane Collaboration (Higgins and Green, 2008) and Preferred Reporting Items for Systematic Reviews and Meta-Analyses Statement guidelines (Moher et al., 2009). The authors have completed the Preferred Reporting Items for Systematic Reviews and Meta-Analyses reporting checklist (Supplementary Material).

### Study Selection

In this meta-analysis, we included studies that investigated cognitive performance in a CP population and compared this performance with that of healthy controls. Two coauthors (Xueying Zhang and Qi Zhao) independently reviewed the titles and abstracts of the retrieved citations. First, we excluded studies that were not investigating the association of pain and cognitive decline. The discrepancy between these two coauthors was reviewed by another author (Rui Gao). Then, full texts of the selected citations were assessed independently for inclusion criteria by the same coauthors and using the same strategy.

We included randomized clinical trials, systematic reviews, and meta-analyses. We excluded editorials, case reports, and descriptive and cross-sectional studies. The definition of cognitive decline could be reported by incident cognitive impairment (binary outcomes) or a decline in cognitive performance (continuous outcomes). For pain assessment, we included studies reporting any kind of pain, except experimental pain.

### Data Extraction

Two coauthors (Xueying Zhang and Changteng Zhang) independently read and extracted data from the included full-text citations. For each study included, we extracted this information: study publication year, geographic location where the study was performed, numbers, type of pain, age, and sex distribution of each group, mean age of participants, and number of female participants.

### Risk of Bias Assessment

We constructed a risk of bias form based on relevant items from the Cochrane Collaboration risk of bias tool and relevant forms of bias relating to case–control study designs (Berryman et al., 2013). Two coauthors (Xueying Zhang and Qi Zhao) completed the bias of each study. Any disagreements were resolved through discussion or by the inclusion of a third reviewer (Rui Gao).

### Data Analysis

Cognitive outcome data were divided according to different cognitive diagnosis methods. We used the mean cognitive outcome data and the standard deviations from each group. The standardized mean difference (SMD) of continuous data or odds ratio (OR) of binary data and 95% confidence intervals (CI) was performed for each cognitive decline outcome. *I*^2^-values were assessed to quantify the heterogeneities. Data for each cognitive decline construct were pooled when results were available from at least two studies. Studies were excluded from the analysis if they did not have sufficient data.

## Results

### Characteristics of the Included Studies

**Figure 1:**
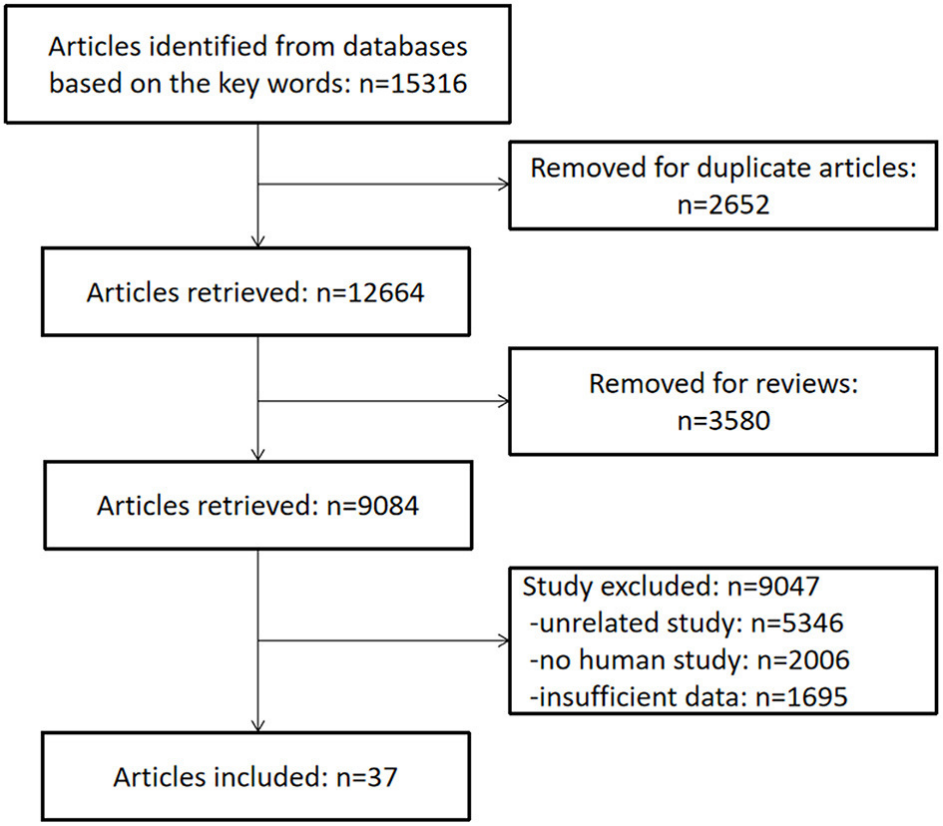
Flow diagram of the literature search.

From the 15,316 records identified by the search methods, 37 studies (Demirci and Savas, 2002; Weiner et al., 2006; Meeks et al., 2008; Shega et al., 2010, 2012; Jonsson et al., 2011; Walteros et al., 2011; Ko et al., 2013; Pirrotta et al., 2013; Butterworth et al., 2014; Docking et al., 2014; Hagen et al., 2014; Martinsen et al., 2014; Pickering et al., 2014; Coppieters et al., 2015; Meeus et al., 2015; Öncü et al., 2015; Dos Santos Ferreira et al., 2016; Fernández-Lao et al., 2016; Santangelo et al., 2016; Schepker et al., 2016; Kaiho et al., 2017; Tzeng et al., 2017; Whitlock et al., 2017; Jordan et al., 2018; Ojeda et al., 2018; Pidal-Miranda et al., 2018; Qu et al., 2018; Van Der Leeuw et al., 2018b, 2020; Veronese et al., 2018; Gu et al., 2019; Ikram et al., 2019; Samartin-Veiga et al., 2019; Kotb et al., 2020; Latysheva et al., 2020; Nakai et al., 2020) were identified, a total of 52,373 patients with CP and 80,434 healthy control participants. These 37 studies included different types of CP: headache (*n* = 7); fibromyalgia syndrome (FM) (*n* = 6); low back pain (*n* = 3); whiplash-associated disorder (*n* = 2); foot pain (*n* = 2); cancer pain (*n* = 1); low back pain and knee pain (*n* = 1); neuropathic pain (*n* = 1); musculoskeletal pain, neuropathic pain, and FM (*n* = 1); post-herpetic neuralgia (*n* = 1); non-cancer pain (*n* = 1); and other unclassified CP (*n* = 13). Among these 37 studies, there were two studies that have invested two kinds of CP, so the total number is 39. Of these 37 studies, cognitive decline was evaluated by different methods: Mini-Mental State Examination (MMSE) (*n* = 6), the short-form 36 health survey questionnaire (SF-36) mental component (*n* = 6), Montreal cognitive assessment (MOCA) (*n* = 4), International Classification of Diseases and Related Health Problems (ICD) classification (*n* = 4), Wechsler Adult Intelligence Scale (WAIS) (*n* = 4), performance validity testing (PVT) (*n* = 2), operation span (OSPAN) (*n* = 2), Repeatable Battery for the Assessment of Neuropsychological Status (RBANS) memory component (*n* = 2) (Table 1), Stroop test (*n* = 1), Memory Failures of Everyday (MFE-30) test (*n* = 1), Syndrom Kurz Test (SKT) (*n* = 1), Cognitive Performance Scale minimus data set (MDS) (*n* = 1), Telephone Interview for Cognitive Status (TICS) (*n* = 1), short test of mental status (STMS) (*n* = 1), and Cambridge Neuropsychological Test Automated Battery (CNTAB) (*n* = 1). Figure 1 describes the process of the study selection. Table 1 describes the characteristics of the included studies.

**Table 1:** Characteristics of the included studies.

**Study**	**Geographic location**	**Pain type**	**Participants**					
			**Chronic pain group**			**Healthy control group**		
			***N***	**Age**	**Gender**	**N**	**Age**	**Gender**
Butterworth2014	Australia	FP	27	49.5 ± 8.7	22F	35	46.9 ± 8.8	27F
Coppieters2015	Belgium	WAD	16	41.6 ± 11.4	13F	22	38.0 ± 13.9	14F
		FM	21	44.5 ± 29.4	16f	22	38.0 ± 13.9	14F
Demirci2002	Turkey	headache	22	45.0 ± 8.6	21F	23	45 ± 8.6	21F
		LBP	23	47.6 ± 12	22F	23	45 ± 8.6	21F
Docking2014	UK	LBP	45	46.9 ± 11.9	34F	45	45.1 ± 10.4	34F
Dos Santos2016	Brasil	CP	45	N/A	N/A	45	N/A	N/A
Fernández2016	Spain	FP	22	47.9 ± 11.0	11F	22	47.2 ± 11	11F
Gu2019	China	CP	25	39.9 ± 9.9	11F	32	33.6 ± 8.7	9F
Hagen2014	Norway	headache	21,871	46.2 ± 15.2	14,063F	29,988	52.3 ± 17.7	13,944F
Ikram2019	USA	CP	6,379	N/A	N/A	12,504	N/A	N/A
Jonsson2011	Denmark	CP	62	52.2	35F	64	51.8	45F
Jordan2018	New Zealand	CP	5,287	82.48 ± 7.48	N/A	36,172	82.48 ± 7.48	N/A
Kaiho2017	Japan	CP	10,702	73.93 ± 5.86	4,730F	3,000	73.2 ± 6	1,858F
Ko2013	Korea	CAP	21	66.1 ± 11.4	8F	48	64.1 ± 13.1	10F
Kotb2020	Saudi Arabia	headache	100	35.31 ± 6.95	60F	105	35.51 ± 7.35	63F
Latysheva2020	Russia	headache	144	42.5 ± 3.17	132F	44	37 ± 5.5	40F
Martinsen2014	Sweden	FM	29	49.8 ± 9.75	N/A	31	46.3 ± 10.7	N/A
Meeks2008	USA	CP	92	79.4 ± 6.8	56F	56	81 ± 6.1	33F
Meeus2015	Belgium	WAD	15	41.6 ± 11.4	3F	16	40.9 ± 13.4	6F
Hirase2020	Japan	LBP,KP	421	75.79 ± 6.5	288F	368	73.65 ± 5.8	218F
Ojeda2017	Spain	NP,MP,FM	254	47.42 ± 8.8	N/A	72	40 ± 11.11	N/A
Öncü2015	Turkey	FM	86	32.3 ± 6.0	N/A	75	32.1 ± 7.7	N/A
Pickering2013	France	PHN	42	72 ± 8	N/A	42	72 ± 8	N/A
Pidal-Miranda2018	Spain	FM	38	47.71 ± 9.63	N/A	33	47 ± 9.01	N/A
Pirrotta 2013	Switzerland	NP	8	61.2 ± 13.2	N/A	9	60.6 ± 14.6	N/A
Qu2017	China	headache	51	37.6 ± 12.6	31F	28	33.9 ± 11.5	18F
Samartin2019	Spain	FM	18	43.9 ± 7.6	22F	22	45.1 ± 7.2	22F
Santangelo2016	Italy	headache	72	34.9 ± 11.2	63F	72	33.8 ± 11.9	66F
Schepker2016	USA	CP	146	75.5 ± 7.23	108F	284	77.1 ± 16.84	183F
Shega2010	Canada	Non-CAP	1,813	79.9 ± 6.0	1,222F	3,273	79.4 ± 6.1	1,817F
Shega2012	Canada	CP	1,332	79.4 ± 5.8	922F	2,435	78.9 ± 5.8	1,359F
Tzeng2017	Taiwan	headache	3,630	N/A	2,463F	10,860	N/A	7,389F
vander Leeuw2018	USA	CP	285	N/A	N/A	156	N/A	N/A
vander Leeuw2019	USA	CP	692	74.38 ± 6.59	480F	2,552	74.29 ± 6.81	1,430F
Veronese2018	Italy	CP	2,317	65.5 ± 9.5	1,244F	4,198	64.2 ± 9.7	2,674F
Walteros2011	Spain	FM	15	50.4 ± 4.6	N/A	15	49.0 ± 6.7	N/A
Weiner2006	USA	LBP	163	73.6 ± 5.2	80F	160	73.5 ± 4.8	66F
Whitlock 2017	USA	CP	1,120	73.8 ± 5.4	851F	8,945	73.6 ± 5.2	5,197F

*FP, foot pain; WAD, Whiplash-associated disorder; FM, Fibromyalgia syndrome; LBP, low back pain; CP, chronic pain; CAP, cancer pain; KP, knee pain; NP, neuropathic pain; MP, musculoskeletal pain; PHN, postherpetic neuralgia; UK, United Kingdom; USA, United States of America; N, number; F, female; N/A, not applicable*.

Only when at least two articles used the same method can be used for meta-analysis. Also, as for WAIS, the four studies used different parts of WAIS, so they could not be analyzed together. Furthermore, for SF-36, mental health and mental component summary are considered as two comparisons. Effects were expressed as SMD for continuous data and OR for binary data, and 95% CIs were given for each study. Weights are from the random-effects analysis.

### Risk of Bias of Included Studies

All studies were deemed to have a risk of bias. One hand is owing to the lack of blinding of the outcome assessors and patients. On the other hand, the bias of these studies was mainly in whether psychiatric disorders were screened for, sample sizes calculated *a priori* (Table 2).

**Table 2:** Risk of bias of included studies.

**Study (ref.)**	**Are cases represe-ntative?**	**Were initial numbers recorded?**	**Were cases diagnosed according to accepted criteria?**	**Were controls using the same diagnostic criteria?**	**Were psychiatric disorders screened for?**	**Were outcome assessors blinded to group status?**	**Were sample sizes calculated a priori?**	**Were confounding variables controlled for?**	**Was subgroup evaluation appropriate?**	**Were there any missing data?**	**Appropriate methods to deal with missing data?**	**Were all outcomes and groups reported?**	**Are the cognitive tests used valid?**	**Are the cognitive tests used reliable?**
Butterworth2014	Y	Y	Y	Y	N	?	N	Y	Y	Y	Y	Y	Y	Y
Coppieters2015	Y	Y	Y	Y	Y	?	N	Y	Y	N	N	Y	Y	Y
Demirci2002	Y	Y	Y	Y	Y	?	N	Y	Y	Y	Y	Y	Y	Y
Docking2014	Y	Y	N	Y	Y	?	N	Y	Y	Y	Y	Y	Y	Y
Dos Santos2016	Y	Y	Y	Y	Y	?	N	Y	Y	Y	Y	Y	Y	Y
Fernández2016	Y	Y	Y	Y	N	?	N	Y	Y	N	N	Y	Y	Y
Gu2018	Y	Y	Y	Y	Y	?	N	Y	Y	Y	Y	Y	Y	Y
Hagen2013	Y	Y	Y	Y	N	?	N	Y	Y	Y	Y	Y	Y	Y
Ikram2019	Y	Y	Y	Y	Y	?	Y	Y	Y	N	N	Y	Y	Y
Jonsson2011	Y	Y	Y	Y	N	?	N	Y	Y	Y	Y	Y	Y	Y
Jordan2018	Y	Y	Y	Y	N	?	N	Y	Y	Y	Y	Y	Y	Y
Kaiho2017	Y	Y	Y	Y	N	?	N	Y	Y	Y	Y	Y	Y	Y
Ko2013	Y	Y	Y	Y	Y	?	N	Y	Y	N	N	Y	Y	Y
Kotb2020	Y	Y	Y	Y	Y	?	N	Y	Y	N	N	Y	Y	Y
Latysheva2020	Y	Y	Y	Y	Y	?	N	Y	Y	N	N	Y	Y	Y
Martinsen2014	Y	Y	Y	Y	Y	?	N	Y	Y	N	N	Y	Y	Y
Meeks2008	Y	Y	N	Y	Y	?	N	Y	Y	Y	Y	Y	Y	Y
Meeus2015	Y	Y	Y	Y	Y	?	N	Y	Y	N	N	Y	Y	Y
Hirase2020	Y	Y	N	Y	Y	?	N	Y	Y	N	N	Y	Y	Y
Ojeda2017	Y	Y	Y	Y	Y	?	N	Y	Y	N	N	Y	Y	Y
Öncü2015	Y	Y	Y	Y	Y	?	N	Y	Y	N	N	Y	Y	Y
Pickering2013	Y	Y	Y	Y	Y	?	N	Y	Y	N	N	Y	Y	Y
Pidal-Miranda2018	Y	Y	Y	Y	Y	?	N	Y	Y	N	N	Y	Y	Y
Pirrotta 2013	Y	Y	Y	Y	Y	?	Y	Y	Y	Y	Y	Y	Y	Y
Qu2017	Y	Y	Y	Y	Y	?	N	Y	Y	N	N	Y	Y	Y
Samartin2019	Y	Y	Y	Y	Y	?	N	Y	Y	N	N	Y	Y	Y
Santangelo2016	Y	Y	Y	Y	Y	?	N	Y	Y	N	N	Y	Y	Y
Schepker2016	Y	Y	Y	Y	Y	?	N	Y	Y	N	N	Y	Y	Y
Shega2010	Y	Y	Y	Y	Y	?	N	Y	Y	N	N	Y	Y	Y
Shega2012	Y	Y	Y	Y	Y	?	N	Y	Y	N	N	Y	Y	Y
Tzeng2017	Y	Y	Y	Y	Y	?	N	Y	Y	Y	Y	Y	Y	Y
vander Leeuw2018	Y	Y	Y	Y	Y	?	N	Y	Y	Y	Y	Y	Y	Y
vander Leeuw2019	Y	Y	Y	Y	Y	?	N	Y	Y	Y	Y	Y	Y	Y
Veronese2018	Y	Y	N	Y	Y	?	N	Y	Y	Y	Y	Y	Y	Y
Walteros2011	Y	Y	Y	Y	Y	?	N	Y	Y	Y	Y	Y	Y	Y
Weiner2006	Y	Y	N	Y	Y	?	N	Y	Y	Y	Y	Y	Y	Y
Whitlock 2017	Y	Y	Y	Y	Y	?	N	Y	Y	Y	Y	Y	Y	Y

*Y, Yes; N, No; ?, unknown*.

### How Cognitive Decline Was Evaluated—Tests and Test Outcomes

Among the 37 included studies, four studies used ICD to identify the cognitive decline diseases, six studies contained 10 comparisons that used SF-36 mental component summary, six studies contained nine comparisons that used MMSE, and four studies used MOCA. In addition, two studies contained three comparisons that used PVT, two studies contained three comparisons that used OSPAN, and two studies used RBANS memory component to evaluate cognition function. Besides these, which can be analyzed, there were 11 studies that used other methods, which could not be analyzed. Four studies contained four comparisons that used different parts of the WAIS scale. Other studies used the Stroop test, MFE-30, SKT, MDS, TICS, STMS, and CNTAB, each contained one comparison.

### Meta-Analysis Outcomes

#### Outcome 1: Results for Short-Form 36 Health Survey Questionnaire Mental Component

**Figure 2:**
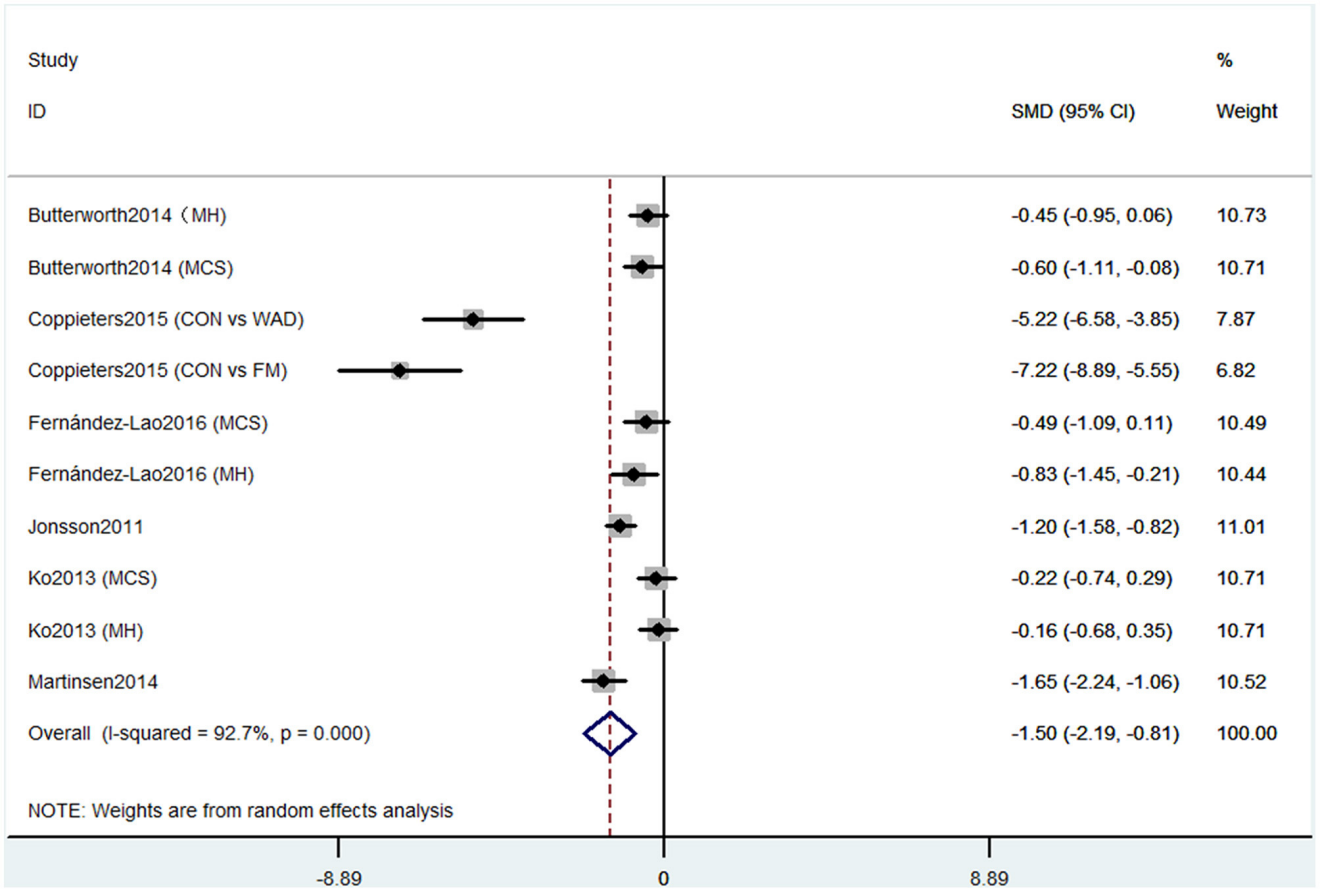
Forest plot of the SMD and 95% CI from a random-effects meta-analysis for the association between chronic pain and incidence of cognitive decline when SF-36 mental component summary was used. MH, mental health score of SF-36; MCS, mental component summary score of sf-36; CON, control group; WAD, whiplash-associated disorder group; FM, fibromyalgia syndrome group.

Short-form 36 health survey questionnaire was used by six studies containing 10 comparisons to evaluate the cognitive decline that mainly refers to mental health. Pooled results of these studies showed that CP was associated with cognitive decline (SMD = −1.50, 95% CI = −2.19 to −0.81). The patients in the pain group were more likely to get the mental problem (Figure 2).

#### Outcome 2: Results for Montreal Cognitive Assessment

**Figure 3:**
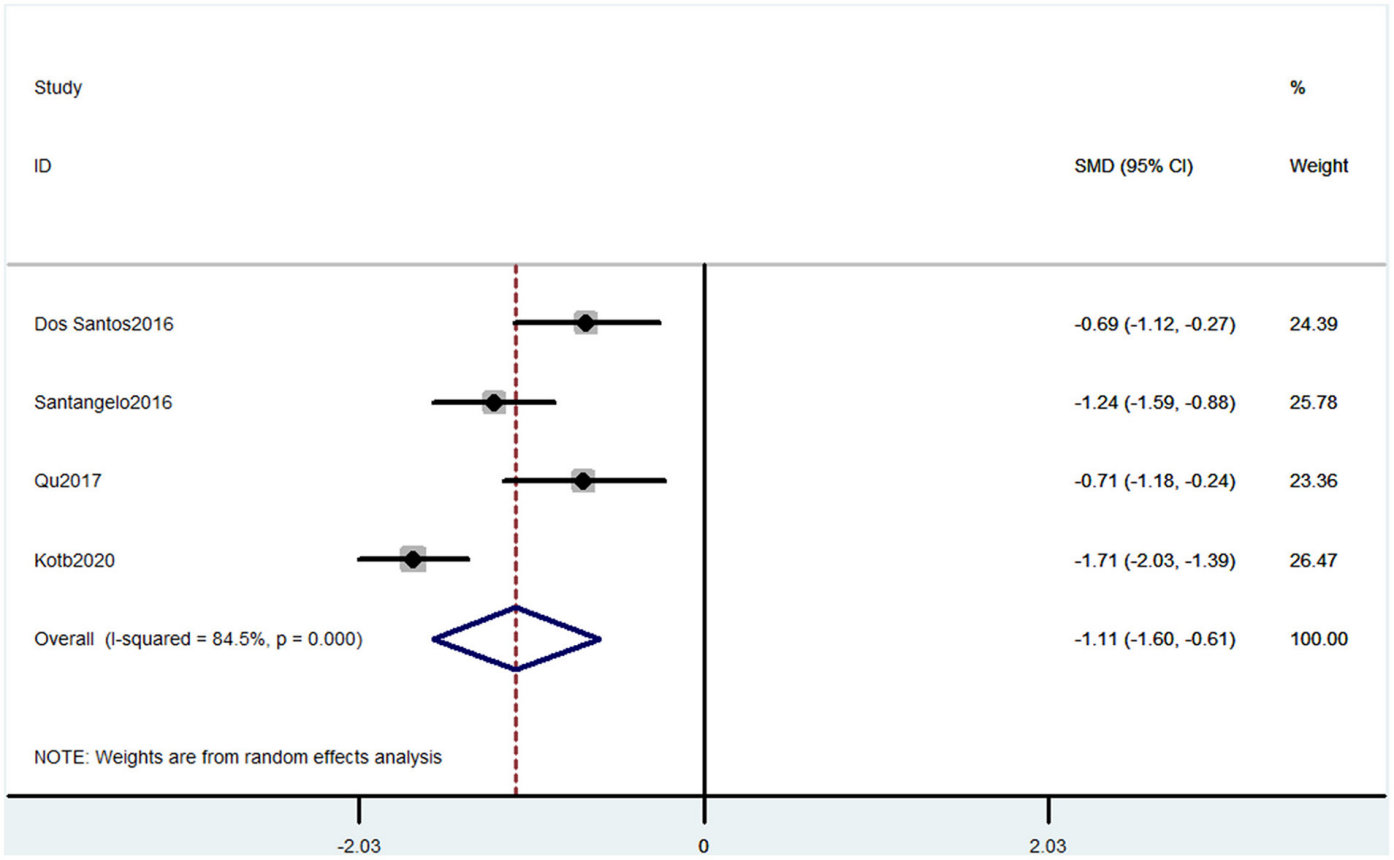
Forest plot of the SMD and 95% CI from a random-effects meta-analysis for the association between chronic pain and incidence of cognitive decline when MOCA was used. The rhombi represent the pooled SMD for this association.

Four studies used MOCA to evaluate cognitive decline. The pooled results of these studies showed that CP was associated with cognitive decline (SMD = −1.11, 95% CI = −1.60 to −0.61). The patients in the pain group were more likely to get low MOCA scores (Figure 3).

#### Outcome 3: Results for Performance Validity Testing

**Figure 4:**
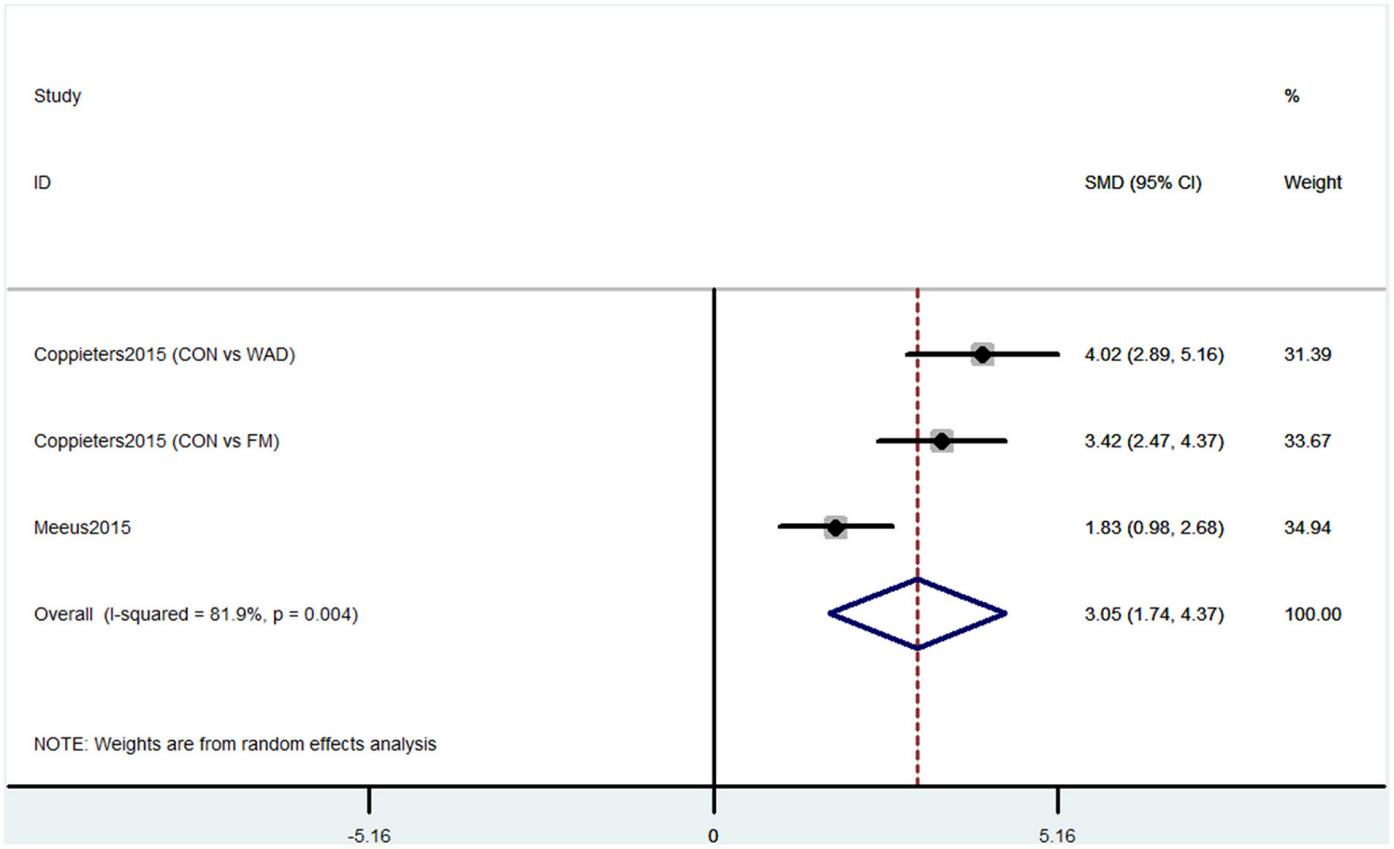
Forest plot of the SMD and 95% CI from a random-effects meta-analysis for the association between chronic pain and incidence of cognitive decline when PVT was used. The rhombi represent the pooled SMD for this association. CON, control group; WAD, whiplash-associated disorder group; FM, fibromyalgia syndrome group.

Performance validity testing was used by two studies containing three comparisons to evaluate the cognitive decline. The PVT has been validated as a measure of sustained attention, alertness, and simple reaction time. The pooled results showed that CP was associated with cognitive decline (SMD = 3.05, 95% CI = 1.74 to 4.37). The patients in the pain group were more likely to have poor cognitive performance (Figure 4).

#### Outcome 4: Results for Operation Span

**Figure 5:**
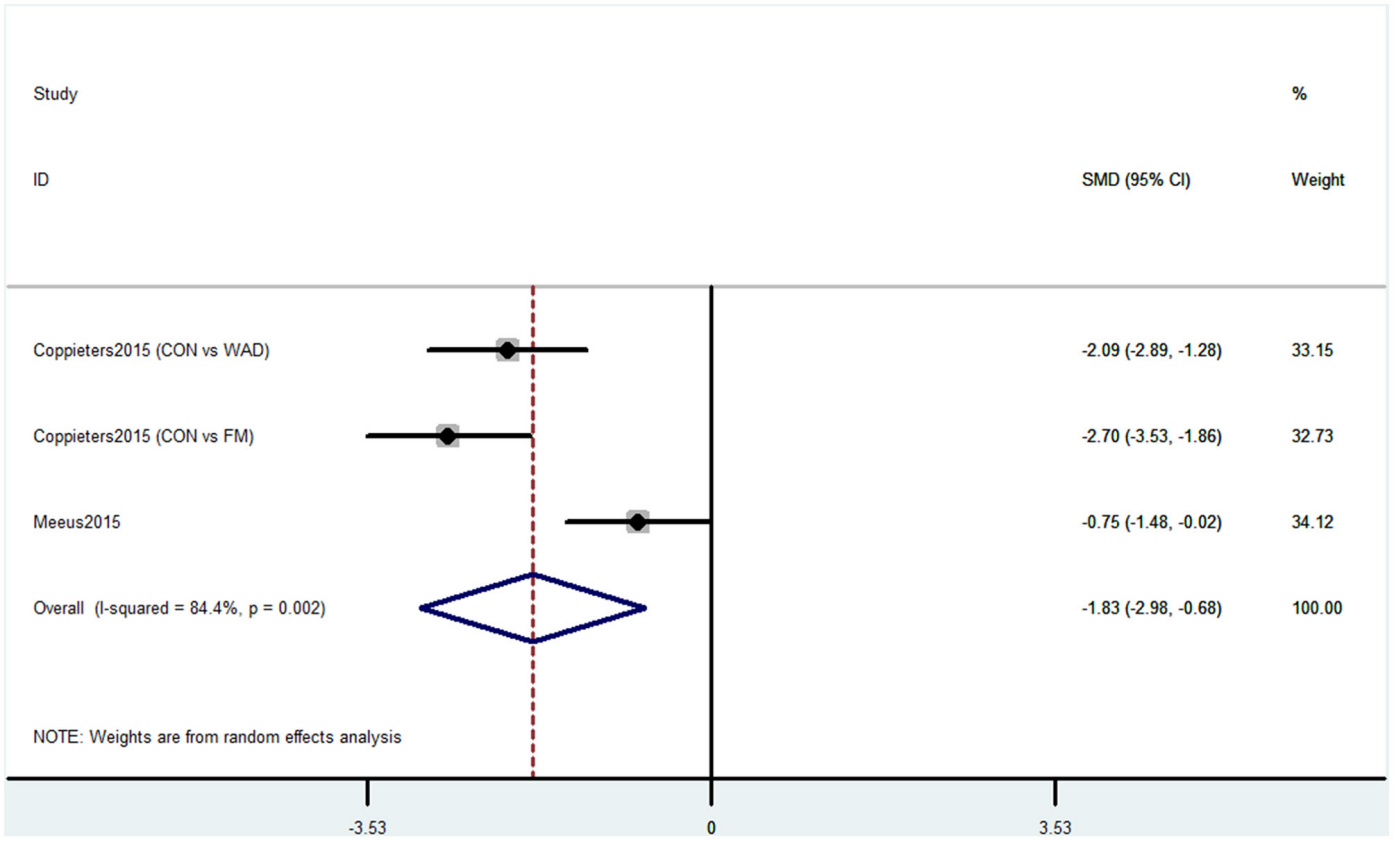
Forest plot of the SMD and 95% CI from a random-effects meta-analysis for the association between chronic pain and incidence of cognitive decline when OSPAN was used. The rhombi represent the pooled SMD for this association. CON, control group; WAD, whiplash-associated disorder group; FM, fibromyalgia syndrome group.

Operation span was used by two studies containing three comparisons to evaluate the cognitive decline. This task was used to assess working memory capacity. The pooled results showed that CP was associated with cognitive decline (SMD = −1.83, 95% CI = −2.98 to −0.68). The patients in the pain group were more likely to have poor working memory performance (Figure 5).

#### Outcome 5: Results for International Classification of Diseases and Related Health Problems

**Figure 6:**
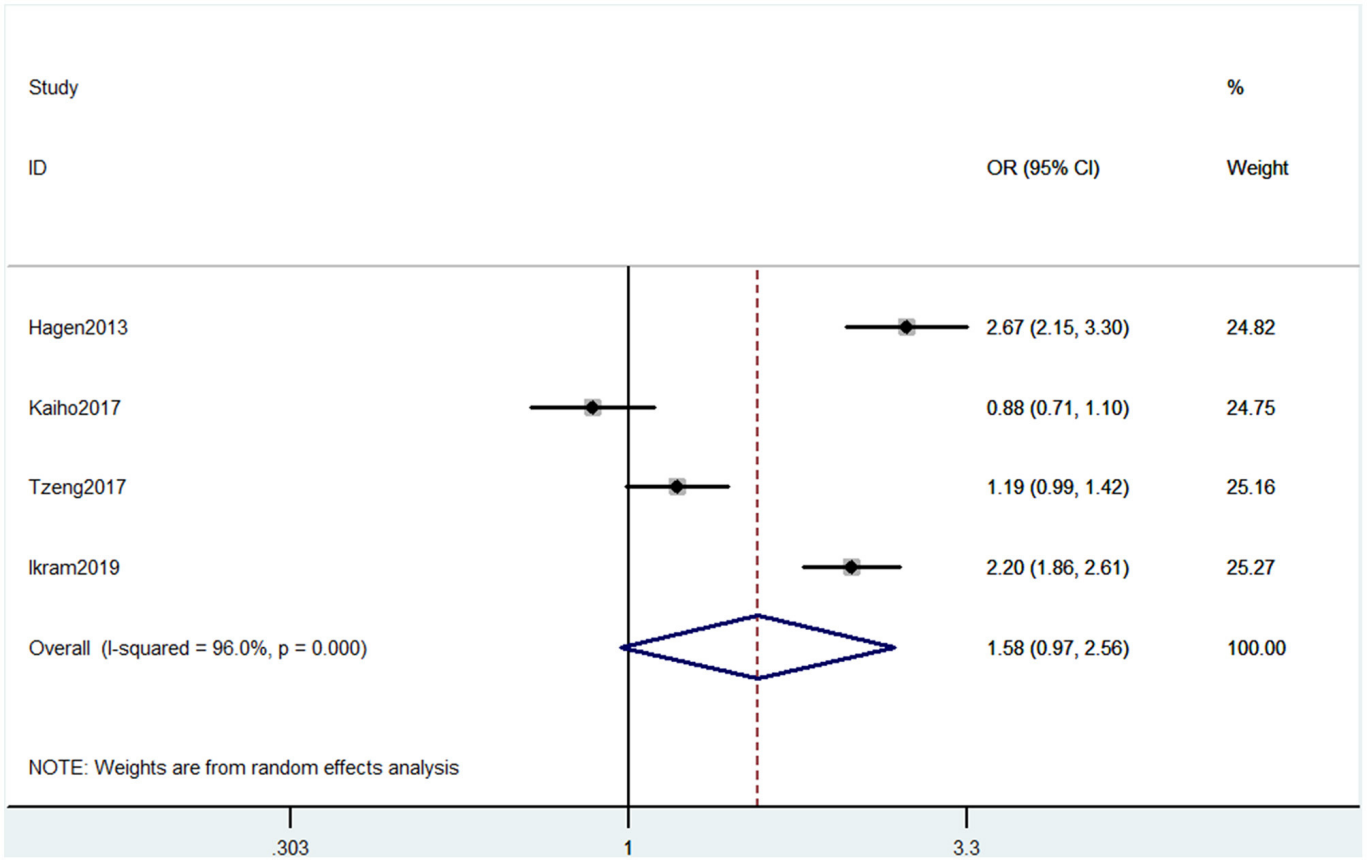
Forest plot of the OR and 95% CI from a random-effects meta-analysis for the association between chronic pain and incidence of cognitive decline when ICD was used. The rhombi represent the pooled OR for this association.

Four studies used ICD to evaluate cognitive decline. The types of cognitive decline defined in these four studies mainly contained dementia and Alzheimer's disease. Pooled results of these studies showed that CP was not associated with cognitive decline (OR = 1.58, 95% CI = 0.97 to 2.56) (Figure 6).

#### Outcome 6: Results for Mini-Mental State Examination

**Figure 7:**
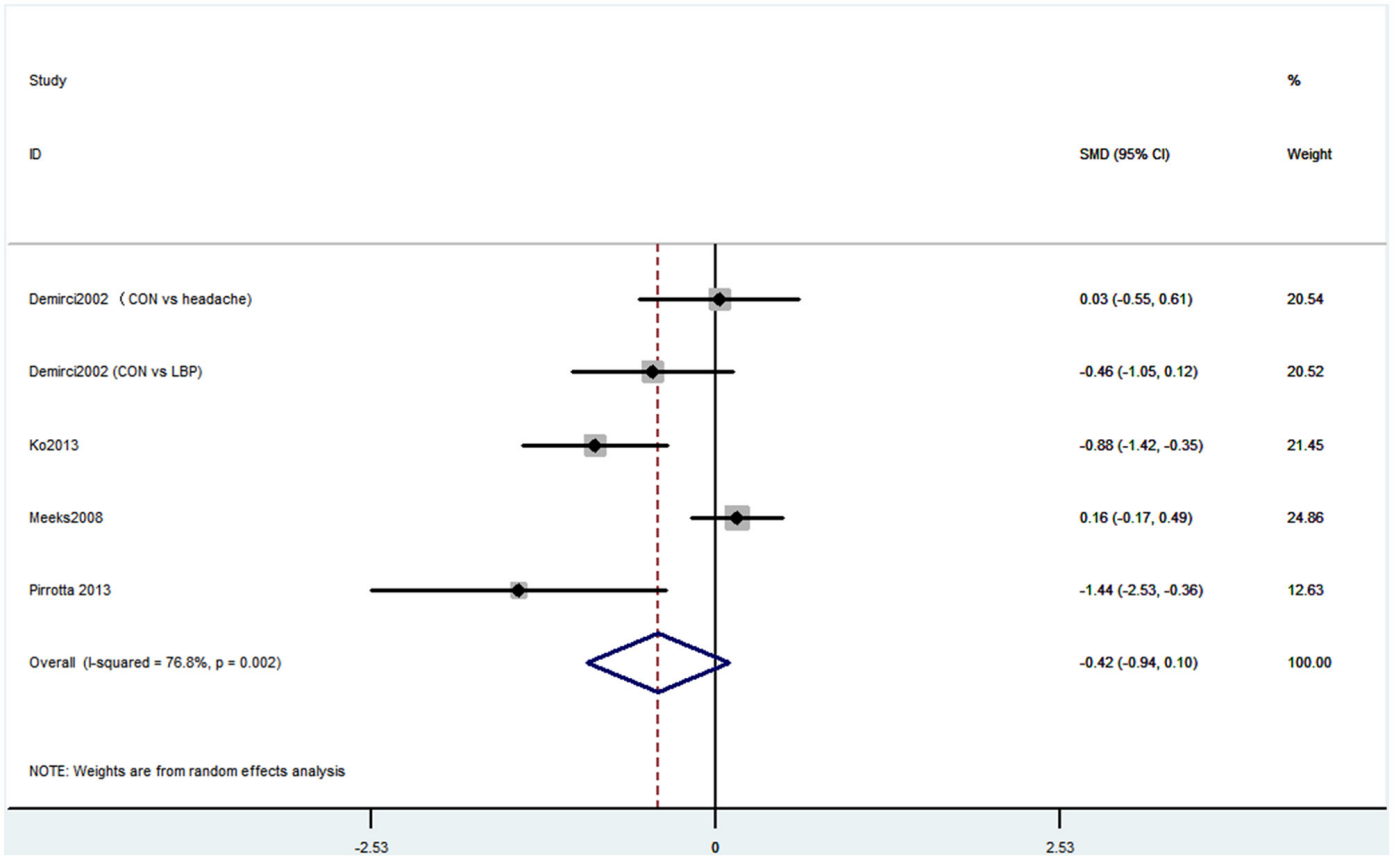
Forest plot of the SMD and 95% CI from a random-effects meta-analysis for the association between chronic pain and incidence of cognitive decline when MMSE continuous score was used. The rhombi represent the pooled SMD for this association. CON, control group; LBP, low back pain.

**Figure 8:**
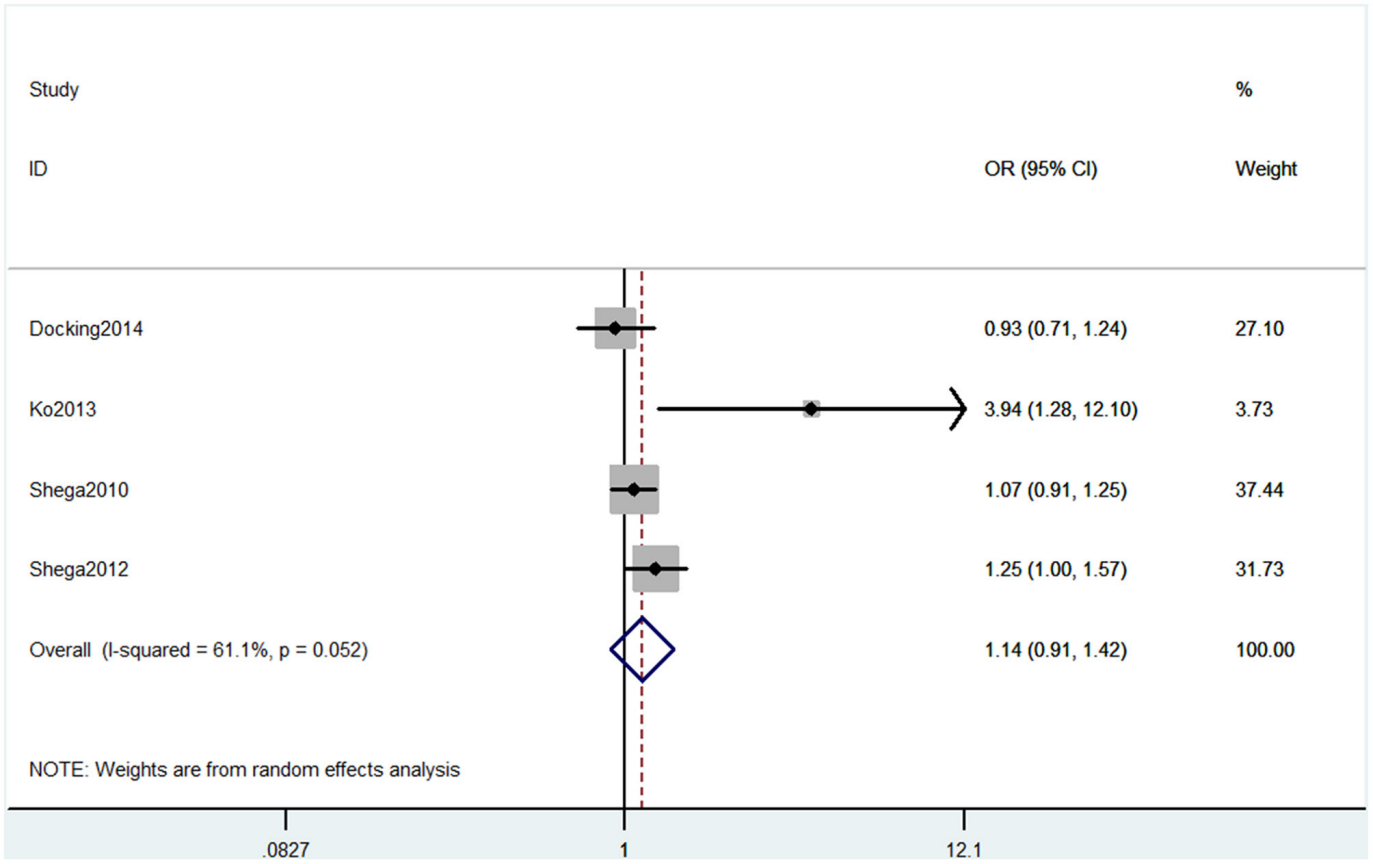
Forest plot of the OR and 95% CI from a random-effects meta-analysis for the association between chronic pain and incidence of cognitive decline when MMSE binary method was used. The rhombi represent the pooled OR for this association.

Six studies used MMSE to evaluate cognitive decline. Among these six studies, four studies containing five comparisons used MMSE primary scores as the results. The pooled results showed that CP is not associated with cognitive decline (SMD = −0.42, 95% CI = −0.94 to 0.10) (Figure 7). Also, three studies contained four comparisons that divided participants into normal cognition and impaired cognition due to the scores. The pooled studies also showed that CP was not associated with cognitive decline (OR = 1.14, 95% CI = 0.91 to 1.42) (Figure 8).

#### Outcomes 7: Results for Repeatable Battery for the Assessment of Neuropsychological Status

**Figure 9:**
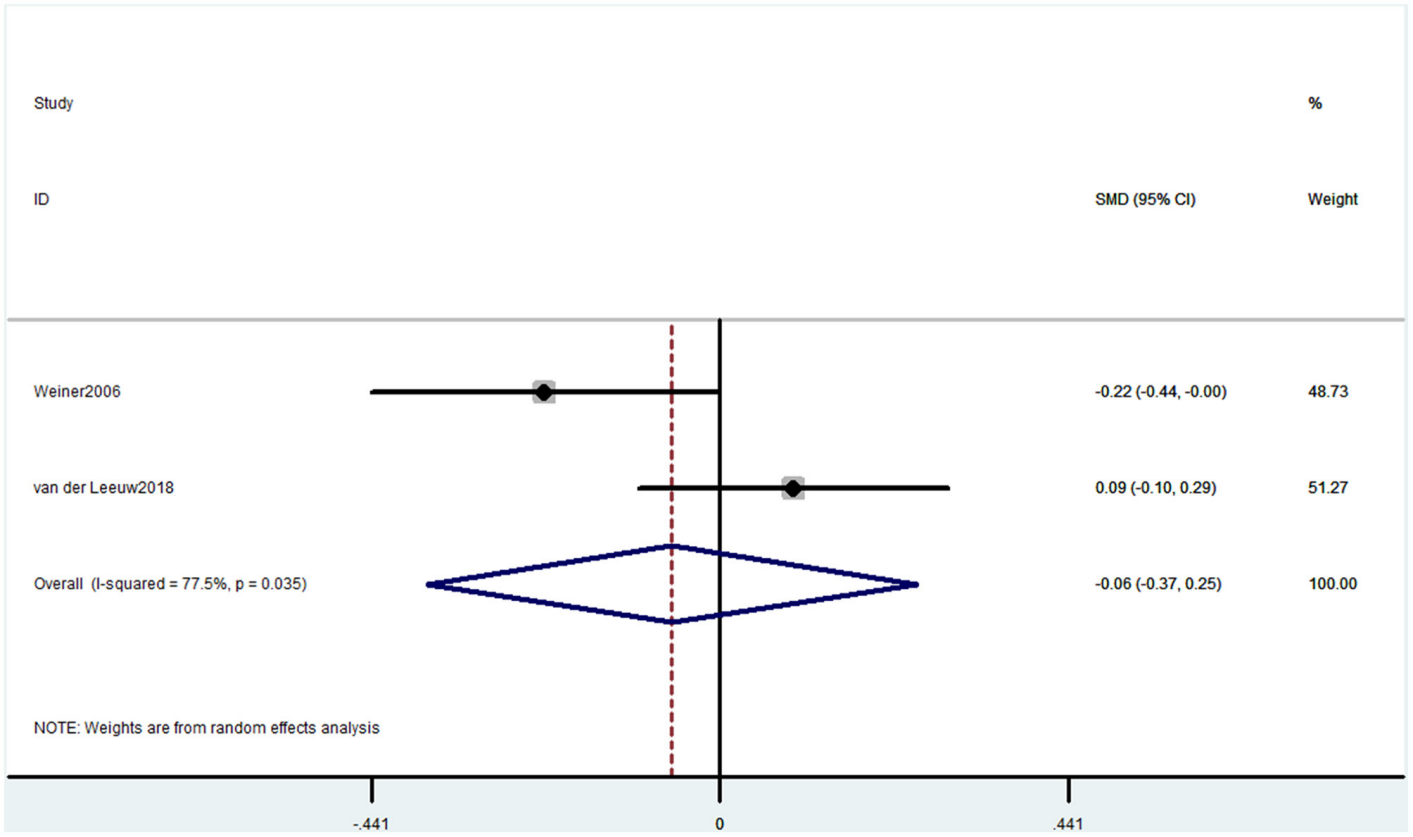
Forest plot of the SMD and 95% CI from a random-effects meta-analysis for the association between chronic pain and incidence of cognitive decline when RBANS was used. The rhombi represent the pooled SMD for this association.

Two studies used the RBANS memory component to evaluate cognitive decline. The pooled results showed that CP was not associated with cognitive decline (SMD = −0.06, 95% CI = −0.37 to 0.25) (Figure 9).

## Discussion

Cognition is a complex concept that includes learning, memory, language, executive function, attention, social cognition, and others (Sachdev et al., 2014). The evaluative method of cognition remains various, making it difficult for researchers to conduct clinical trials or meta-analyses. We performed the analysis due to the different methods for cognitive assessment used in the 37 included articles. Pooled results from the outcomes of our study reflect an opposite result. The results showed a consistent, significant effect between CP and cognitive decline when the methods of SF-36 mental component summary, MOCA, PVT, and OSPAN were used. However, the results provided no evidence for an effect when methods of ICD, MMSE, and RBANS were used.

Numerous studies containing clinical and animal studies have shown CP could result in cognitive decline (Moriarty et al., 2011; Yang et al., 2014; Whitlock et al., 2017; Shiers et al., 2020). For example, Whitlock et al. (2017) found persistent pain was associated with accelerated memory decline and increased probability of dementia based on a cohort study with biennial interviews of 10,065 community-dwelling older adults. This article was published in the *Journal of the American Medical Association Internal Medicine*. Mechanism studies explain pain, and cognitive decline may share some same pathways (Moriarty et al., 2011; Phelps et al., 2021). Also, there have been some meta-analyses exploring the relationship between pain and some types of cognitive decline. For example, one meta-analysis showed CP was associated with memory deficits but only in behavioral outcomes, whereas the physiological effects showed no effect (Berryman et al., 2013). Another meta-analysis focused on executive function showed that people with CP might have impaired executive function (Berryman et al., 2014). However, a recent meta-analysis showed persistent pain was not associated with the incidence of cognitive decline from prospective longitudinal studies (De Aguiar et al., 2020).

Our meta-analysis showed some evidence that CP might be related to cognitive impairment. Six studies containing 10 comparisons used SF-36 that show that CP was associated with cognitive decline. The SF-36 was used to assess physical function, mental health, and health-related quality of life (Brazier et al., 1992). These six studies were from different countries, and the heterogeneity was high. Meanwhile, the pain types of these studies were different, four studies were CP without any subgroups, one study contained both whiplash-associated disorder and FM (Coppieters et al., 2015), and one study only contained FM (Martinsen et al., 2014). These factors together may induce high heterogeneity. Studies using MOCA also showed that CP was associated with cognitive decline. MOCA is used to evaluate the global cognitive status and several cognitive domains: memory, attention, language, orientation, and visuospatial and executive function domains (Nasreddine et al., 2005). These studies also had high heterogeneity. Three studies were performed on headache patients, but one investigated CP without more detailed classification. Thus, more homogeneous controlled clinical trials are needed to confirm the results. The PVT has been validated as a measure of sustained attention, alertness, and simple reaction time. Two studies containing three comparisons used PVT also showed that CP was associated with cognitive decline. However, only two studies were included in this analysis. Therefore, the number of included participants may not be enough, and more studies are needed. Another evidence was that two studies using OSPAN to evaluate memory also verified the association between CP and cognitive decline (Quach et al., 2016). Besides, a study that used SKT also showed that preoperative CP distracted the attention before surgery and reduced the recovery of attention and memory abilities after the surgery in non-elderly patients (Gu et al., 2019). Moreover, animal studies showed that both rats and mice with CP could have cognitive disorders (Owoyele et al., 2021; Zhang et al., 2021).

The association between CP and cognitive decline is still unknown. An important key is that it is difficult to combine all the evaluative methods of cognition. In some methods such as MMSE, the higher the score, the worse the cognitive impairment. Some others, such as SF36, are opposite. In addition, some methods studied different types of cognitive impairment and could not be analyzed together. A total of 37 articles included in our study contained 15 methods that have been listed earlier. The sample size of studies using the same method was different. The study of Hagen et al. (2014) has the most sample size and far exceeded other studies. Another study with a larger sample size is Kaiho et al. (2017). Furthermore, these two studies both used the ICD method. However, the negative result of Kaiho et al. (2017) reversed the positive results of Hagen et al. (2014) and the other two studies in outcome 5 (Figure 6). Also, for other outcomes, whether the sample size is enough to support the positive results is unsure. So the conclusion needs a larger sample size and multicenter studies. Secondly, the evaluative accuracy of these methods is also different. Mini-Mental State Examination and MOCA, as the most basic methods, are regarded as brief screening tools (Nasreddine et al., 2005; Ciesielska et al., 2016). Wechsler Adult Intelligence Scale is also a comprehensive scale for neuropsychological status, including reasoning, processing speed, and working memory (Whipple Drozdick and Munro Cullum, 2011). Short-form 36 health survey questionnaire is a health-related evaluation tool, so the mental component summary and mental health part could only reflect some mental health (Brazier et al., 1992). Short test of mental status was found to be more sensitive than MMSE and can be used by clinicians to differentiate both normal cognition from MCI and MCI from probable Alzheimer's disease (Çebi et al., 2020). Performance validity testing, OSPAN, RBANS, and SKT are used to evaluate some aspects, including memory, attention, and reaction (Meeus et al., 2015; Van Der Leeuw et al., 2018a; Gu et al., 2019; Loring and Goldstein, 2019). Stroop test is used to mainly test reactions (Coppieters et al., 2015). Memory Failures of Everyday is used to assess memory problems (Samartin-Veiga et al., 2019). Minimus data set, TICS, and CNTAB are general scales but used with low frequency (Pickering et al., 2014; Jordan et al., 2018; Van Der Leeuw et al., 2020). Therefore, each of these methods has its own advantages and disadvantages. Hopefully, future studies would compare these methods to confirm their accuracy for investigating.

Our meta-analysis also showed some negative results, possibly due to the cognitive evaluation method adopted. Mini-Mental State Examination was the essential evaluation of cognitive decline. However, only when the cognitive impairment is severe enough, this test works. Also, there have been some studies that suggested MMSE is less accurate than MOCA (Roalf et al., 2013; Pinto et al., 2019). As for RBANS, only two studies were included; the sample size was too small to account for the result. Besides, RBANS is a complex assessment containing many aspects (Weiner et al., 2006), and only one part of the two studies was identical. Also, one recent meta-analysis has found this controversial conclusion between persistent pain and cognitive function among the olds (De Aguiar et al., 2020). However, they did not care about the different cognitive evaluation methods. Therefore, we have a concern about their conclusion. It is important to make it comparable. Our risk of bias assessments has provided some recommendations for future studies. For example, acknowledged diagnostic criteria should be used for CP and cognitive decline. Also, it is critical for the future study to consider the following aspects, such as much more accurate screening of psychiatric disorders, blinded method, and calculation of sample size in advance. In addition, a clinical study of a large sample size should be performed, as many studies included only had small sample sizes.

## Conclusion

In the present study, we had found that CP might be associated with cognitive decline when the cognitive, evaluative scales were used, including the SF-36 mental component summary, MOCA, PVT, and OSPAN. Future studies are needed with a unified cognitive evaluation method to clarify the association between CP and cognitive function.

## Data Availability Statement

The original contributions presented in the study are included in the article/Supplementary Material, further inquiries can be directed to the corresponding author/s.

## Author Contributions

CC and TZ: conception and design. CC: administrative support. XZ, RG, QZ, and CZ: collection and assembly of data. XZ, RG, and HC: data analysis and interpretation. All authors: manuscript writing and final approval of manuscript.

## Funding

This study was funded by the National Natural Science Foundation of China (Nos. 81870858, 81500937, and 82171185 to CC), the National Key R&D Program of China (No. 2018YFC2001800 to TZ), the National Natural Science Foundation of China (No. 81671062 to TZ), China Postdoctoral Science Foundation (Grant No. 2020M673234 to RG), and the Science and Technology Plan Project of Sichuan Province (No. 2020YJ0051 to CC).

## Conflict of Interest

The authors declare that the research was conducted in the absence of any commercial or financial relationships that could be construed as a potential conflict of interest.

## Publisher's Note

All claims expressed in this article are solely those of the authors and do not necessarily represent those of their affiliated organizations, or those of the publisher, the editors and the reviewers. Any product that may be evaluated in this article, or claim that may be made by its manufacturer, is not guaranteed or endorsed by the publisher.
